# Can nonvolatile tastants be smelled during food oral processing?

**DOI:** 10.1093/chemse/bjad028

**Published:** 2023-08-17

**Authors:** Yue He, Jianshe Chen, Weiyao Shi, Jingang Shi, Tian Ma, Xinmiao Wang

**Affiliations:** Laboratory of Food Oral Processing, School of Food Science and Biotechnology, Zhejiang Gongshang University, Hangzhou, China; Laboratory of Food Oral Processing, School of Food Science and Biotechnology, Zhejiang Gongshang University, Hangzhou, China; EPC Natural Products Co., Ltd., Beijing, China; EPC Natural Products Co., Ltd., Beijing, China; Laboratory of Food Oral Processing, School of Food Science and Biotechnology, Zhejiang Gongshang University, Hangzhou, China; Laboratory of Food Oral Processing, School of Food Science and Biotechnology, Zhejiang Gongshang University, Hangzhou, China

**Keywords:** gustation, olfaction, food oral processing, oral–nasal mass transfer

## Abstract

While accumulating evidence implied the involvement of retro-nasal sensation in the consumption of nonvolatile taste compounds, it is still unclear whether it was caused by the taste compounds themselves, and if so, how can they migrate from the oral to nasal cavity. At first, we proposed aerosol particles as an alternative oral–nasal mass transfer mechanism. The high-speed camera approved that aerosol particles could be generated by the typical oral and pharynx actions during food oral processing; while the narrow-band imaging of nasal cleft and mass spectrometry of nostril-exhaled air approved the migration of aerosol within the oral–nasal route. Then, the “smelling” of taste compounds within the aerosol particles was testified. The four-alternative forced choices (4AFC) approved that the potential volatile residues or contaminants within the headspace air of pure taste solution cannot arouse significant smell, while the taste compounds embedded in the in vitro prepared aerosol particles can be “smelled” via the ortho route. The “smell” of sucrose is very different from its taste and the “smell” of quinine, implying its actual olfaction. The sweetness intensity of sucrose solution was also reduced when the volunteers’ noses were clipped, indicating the involvement of retro-nasal sensation during its drinking. At last, the efficiency of aerosol as a mechanism of oral–nasal mass transfer was demonstrated to be comparable with the volatile molecules under the experimental condition, giving it the potential to be a substantial and unique source of retro-nasal sensation during food oral processing.

## Introduction

Retro-nasal sensation plays a key role in our appreciation of food, which is thought to be aroused by the aroma molecules that are released from the food within the oral cavity, due to the aroma molecules’ volatile natures (Small 2012). However, the low or even nonvolatile compounds were also found to play an essential role in the overall perception of food or beverage that could hardly be expected with their volatilities (Frank et al. 2011; Wang et al. 2022a, 2022b). For example, the nonvolatile taste compounds were found to have a strong influence on the intensity of certain aroma molecules in a reconstructed red wine (Frank et al. 2011); adding lactic acid in baijiu significantly decreased the olfactory thresholds of ethyl lactate and ethyl acetate (Wang et al. 2022b).

Even the consumption of pure nonvolatile taste solution was found to arouse the retro-nasal sensation. Healthy subjects experience increased thresholds for taste recognition and detection (Tsuji et al. 2018) or reduced perception of taste intensity after induced nasal obstruction (Mojet et al. 2003, 2005; Smutzer et al. 2018). Gustatory dysfunctions are frequently identified in patients with chronic rhinosinusitis (Xie et al. 2021) and were also found to be improved after the nasal obstruction was surgically improved (Tsuji et al. 2018). The mice with impaired olfaction also exhibited severely blunted responsiveness toward sugars, despite the intact T1R2 and T1R3 receptors they have (Schier et al. 2019). These observations show that blockage or damage of the retro-nasal sensation can also influence the perception of nonvolatile taste compounds that were originally considered odorless and inaccessible to the olfactory receptor. This leads us to speculate that molecule is not the sole mass transfer mechanism for retro-nasal sensation during eating or drinking, and the nonvolatile compounds can also be detected by the olfactory receptors via a very different mass transfer mechanism.

Aerosol is a suspension of liquid or solid particles in the gas, with particle diameters in the range of 0.01–100 µm. Inspired by the mass production of bio-aerosol during human respiratory activities (Wang et al. 2021), we hypothesized that the retro-nasal sensation of orally ingested nonvolatile taste compounds was caused by the aerosol generated during food oral processing. This study was therefore structured to answer 3 immediate and critical questions regarding the plausibility of this hypothesis: (i) can the aerosol particles be generated and migrate within the oral–nasal cavity during food oral processing; (ii) can the nonvolatile taste compounds be “smelled” in the form of aerosol; (iii) can the aerosol be a comparable oral–nasal mass transfer mechanism?

## Experiment 1: generation and migration of aerosol during drinking

### Method

#### Study design

**Fig. 1.**
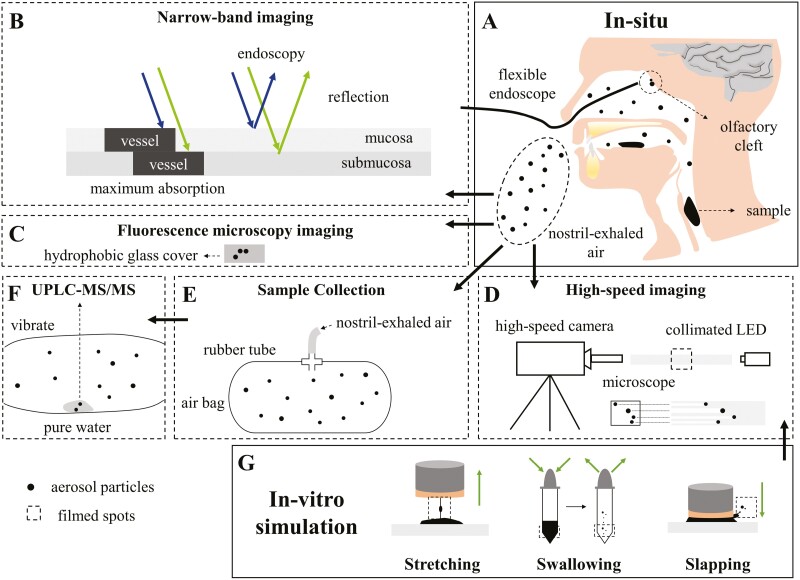
Schematic illustration of experiment 1. A) Schematics of in situ experiments that the flexible endoscope was positioned within the nostril or the nostril-exhaled air was collected during drinking. B) Principles of NBI that lasers are absorbed by vessel, making it darker than the its surrounding tissue. C) Fluorescence imaging of glass cover with the nostril-exhaled air deposited. D) Experimental setting-ups for high-speed imaging. A microscope was attached to a high-speed camera to film the small aerosol particles. The collimated LED entered the microscope at an angle of 180°. E) The settings of airbags used to collect air sample. F) UPLC–MS/MS analysis of nostril-exhaled air. G) In vitro simulation of 3 typical oral and pharyngeal activities during food oral processing.

Human activities, especially respiratory activities have been approved to be efficient aerosol-generating procedures (Wang et al. 2021): speaking, breathing, coughing, and sneezing can create aerosols originating from the sites of the oral cavity, alveoli, bronchiole, bronchus, and larynx (Xu et al. 2022), with the film breakage and surface shearing considered as the 2 major mechanisms of aerosol generation (Morawska et al. 2009; Johnson et al. 2011; Abkarian and Stone 2020; Hamed et al. 2020; Guo et al. 2021; Xu et al. 2022). As a reasonable comparison (Table 1), we tend to believe that swallowing and tongue movements (stretching and slapping) are the major aerosol-generating actions during drinking or eating. To confirm this, 3 in vitro tests (Fig. 1G) were designed to, respectively, mimic fluid stretching, fluid slapping, and bolus flow, and their aerosol-generating processes were observed using a customized high-speed camera (Fig. 1D). The aerosol particles within the volunteer’s nostril-exhaled air were also imaged (Fig. 1A). Due to the limitation of this imaging method (180° light scattering) that only small items within the transparent background can be imaged, the in situ aerosol-generating process were not obtained here. The migration of aerosol particles within the oral–nasal cavity was indirectly approved by the detection of the orally ingested nonvolatile compounds in the olfactory cleft or nostril-exhaled air: the narrow-banding imaging (NBI) of volunteer’s olfactory cleft before or after his consuming of c-phycocyanin (60%, w/w, Zhejiang Binmei Biotechnology Co., Ltd, China) solution was determined to approve the deposition of oral aerosol originated from the oral or pharyngeal cavity (Fig. 1B); the ultra-high performance liquid chromatography (UPLC–MS/MS) (Fig. 1E and F) and fluorescence microscopy imaging (Fig. 1C) of volunteers’ nostril-exhaled air were determined to approve the oral aerosol can migrate from oral or pharyngeal cavity to nasal cavity. To simplify the study, only aqueous systems were included here, without considering other liquid or solid systems and human oral physiology. All procedures followed were in accordance with the ethical standards of the responsible committee on human experimentation (institutional and national) and with the Helsinki Declaration of 1975, as revised in 2008. Informed consent was obtained from all participants in the study.

**Table 1.** Comparison of favorable conditions for the generation of aerosol particles by respiratory activities and activities during food oral processing.

	Respiratory activities	Food oral processing
Interfacial film	Airway lining fluid Composition: 94% water and 6% solids (mucin, ions, and others) Structures: a low viscosity aqueous periciliary fluid (sol layer) adjacent to the lung tissues which enables free ciliary movement and a mucus gel layer which lies atop the periciliary layer and alters the viscoelastic properties of the airway fluid. The thickness is 10 and 30 mm in the trachea and 2 and 5 mm in the bronchi (Hamed et al. 2020) Surface tension: *γ* ∼ 35 mN/m Rheology: ~1 mPa s	Saliva Composition: 98% water + 2% solids (amylase, mucin, ions, and others) Structures: a continuous water phase containing electrolytes and water-soluble substances; a scaffold-like continuous network containing salivary glycoproteins of high molecular weight; macromolecules present at the saliva network core (within the protein filaments) such as less water-soluble proteins; and lipoid material, water-insoluble substances and epithelial cells (Watanabe et al. 2007) Surface tension: *γ* ∼ 50 mN/m Rheology: 1–10 mPa s
Temperature and humidity	Alveolar gas: 37.0 °C, 100% RH Hypopharyngeal: 33.0 °C, ~100% RH	Retro-nasal: 32.0 °C, ~100% RH Oral cavity: 37.0 °C (mouth closed), ~100% RH
Airflow	Quite breathing: ~ 0.4 L/s Cough: 12 L/s, Re = 50,000 (turbulence)	Close to quite breathing at the back of oral cavity
Movement	Re-opening of terminal bronchiole High air velocity passing the closed epiglottis Vocal fold vibration and open/close action	Teeth movement create and break the filaments Tongue movement shear and rupture the interfacial fluids Pharyngeal contraction sequences the food bolus with possible turbulence flow

#### High-speed imaging of nostril-exhaled air and in vitro aerosol generation

A high-speed camera (PCO.dimax HD, PCO AG, Germany) attached to the microscope was used to record the aerosol-producing procedures. The magnification of the microscope was 360. The region of interest was 672 × 484 with an exposure time of 4 µs and a frame-per-second of 10,000. To decouple the depth of focus and the magnification of the microscope, the collimated LED entered the microscope at an angle of 180°. The experimental setting-up and imaging principle are illustrated in Fig. 1D. Three oral movements were simulated (Fig. 1G): a cylindrical metal covered with porcine tongue skin (purchased from the local farmers’ market in Hangzhou, China) was detached at the speed of ~15 mm/s from the surface of polydimethylsiloxane (PDMS) to mimic the stretching of the tongue inside the oral cavity; a cylindrical metal covered with porcine tongue skin banged onto the surface of PDMS at the speed of ~3.5 m/s to mimic the slapping of the tongue inside the oral cavity; a dropper with an external diameter of 7 mm was squeezed and released to mimic the airflow created by the swallowing. A 5-mL 300 mM sucrose (99.5%, w/w, Aladdin Inc., China) solution was swirled within the oral cavity for 5 s and then spat out as the testing solution. The testing solution for each test was always collected from the same subject after rinsing his mouth with the bottled pure water (Wahaha Inc., China). The in situ produced aerosols (Fig. 1A and D) were also filmed: one subject was required to exhale the air into the imaging zone after swallowing a 5-mL 300 mM sucrose solution. At least 3 replicates were done for the 3 simulating and in situ tests.

#### NBI of olfactory cleft

NBI of the olfactory cleft was conducted at the endoscopy center of Ninghai First Hospital with Olympus AF 290 (Olympus Corporation, Japan). One healthy subject with no nasal obstruction was recruited for the test. Bright-field imaging was conducted right before the NBI to assure the subject meet the criteria. During the NBI, the subject sits comfortably with the flexible endoscope carefully introduced into his nostril. Images were obtained when the subject hold the c-phycocyanin solution (800 mg/L) in his mouth after swirling for 5 s and swallowed it. The experimental procedure is illustrated in Fig. 1A and B. At least 3 replicates were done with a time interval of 1 h.

#### Fluorescence microscopy imaging of nostril-exhaled air

The hydrophobic glass coverslips were carefully positioned beneath the subject’s nostrils to collect the exhaled air after he swirled and swallowed the c-phycocyanin or riboflavin (98%, Aladdin Inc., China) solution (800 mg/L), the edible and fluorescent nonvolatile compounds. Immediately after collection, the glass coverslips were imaged by a fluorescence microscope (DM3000, Leica Microsystems Inc., Wetzlar, Germany) with the 2-s exposure time, green or blue light laser, and 3.0× gain. The experimental procedure is illustrated in Fig. 1A and C. At least 3 replicates were done.

#### UPLC–MS/MS of nostril-exhaled air

Four healthy subjects (2 males and 2 females, average ages of 23.8 ± 0.5, body mass index [BMI] of 20.9 ± 1.7) with no nasal obstruction were recruited, and were asked to abstain from eating the night before collection, and rinsed their mouth with bottled pure water right before the collection, to minimize the effects of oral and gastrointestinal residues. The collection started at 08:00 and finished within 60 min. During collection, subjects naturally exhaled the air into the airbags for once immediately after their finish of required oral behaviors. Two oral behaviors were used to minimize or maximize the aerosol production: holding the 5 mL sample within the mouth for 5 s and spitting them out (labeled as H); swirling the 5 mL sample within the mouth for 5 s and swallowing them (labeled as S). Three samples were tested: bottled pure water (labeled as W), 300 mM (labeled as S1), and 600 mM (labeled as S1) sucrose solution. The collecting procedure was carried out 10 times for each condition and sample with the same airbags to obtain a sufficient amount of sucrose. After collection, 1.5 mL of bottled pure water was injected into the airbags, gently vibrated for 24 h, and then removed for UPLC–MS/MS analysis (see Supplementary Information for details). One blank sample (ambient air and bottled pure water were injected in airbags) were also collected and analyzed in each collection, to assure the reliability of this collection procedure.

#### Statistics and significance

The UPLC–MS/MS results were analyzed among treatments (Fig. 3A) or subjects (Fig. 3B). The homogeneity of variances is not achieved among treatments, and hence their significances were determined using ANNOVA Dunnett T3’ post hoc tests; while homogeneity of variances is achieved among subjects, and hence their significances were determined using ANNOVA Scheffe’s post hoc tests. All the statistical tests were conducted using SPSS 26.0.0.0.

**Fig. 2.**
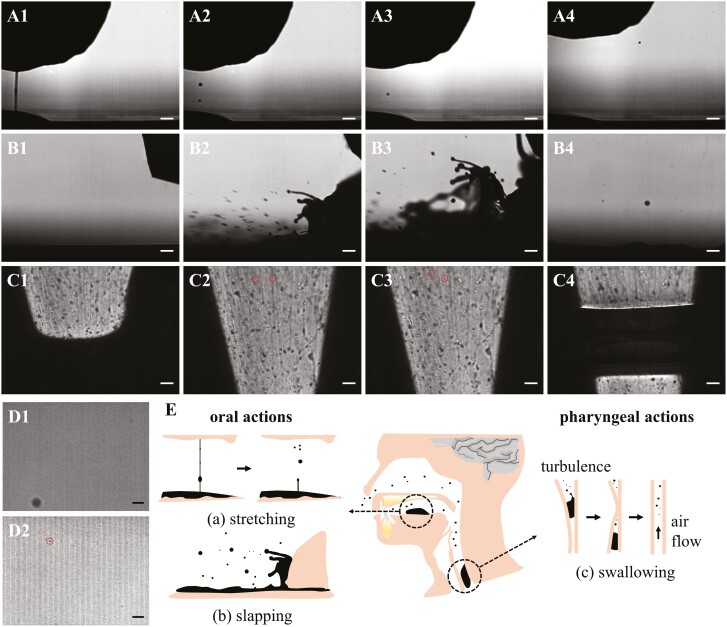
The generation of aerosol particles during drinking. Aerosol generation during the stretching between porcine tongue and PDMS surface in 1 trial, consisting of filament formation (A1), aerosol formation (A2), and migration (A3 and A4). Aerosol generation during the slapping between porcine tongue and PDMS surface in 1 trial, consisting of pre-impaction (B1), impaction (B2 and B3), and aerosol floating (B4). Aerosols generation during the squeeze and release of dropper in 1 trial, consisting of squeezing (C1), aerosol flowing with the air backflow (C2 and C3), and backflow of bubble (C4). D1 and D2) Aerosols in the mouth-exhaled air after the subjects swirled and swallowed 5 mL of 300 mM sucrose solution. E) Schematic illustration of the aerosol-producing mechanisms during food oral processing. Scale bar, 100 µm. Circles were used to highlight aerosols. See Supplementary Movies S1–S4.

### Results

#### The generation of oral aerosol particles during drinking

In the film breakage mechanism of bio-aerosol generation, the creation and breakage of thin films are of critical importance (Hamed et al. 2020), which can be achieved by stretching, splashing, and slapping. These movements are quite common during the handling of food or beverage within the mouth and swallowing them. A typical filament containing beads was formed (Fig. 2A1) as the porcine tongue surface detached from the PDMS surface, and fractured to form aerosol particles (Fig. 2B2) when further stretched. The formed particles either re-immersed into the substrate fluid (Fig. 2B2 and B3) or flew away (Fig. 2B4) into the air to form an aerosol and a larger number of much smaller aerosol particles formed during the breakup of thinner filaments (Supplementary Movie 1). In the real scenario, the presence of papillae and roughness of the human tongue could potentially form tremendous thin filaments and their breakage might also result in a large amount of aerosol particles once the tongue separates from e.g. hard palate and/or teeth. When slapped by the porcine surface, the saliva–sucrose mixture splashed, instantly creating a large number of droplets of varying sizes (Fig. 2B1–B3). Some larger ones return to the substrate due to gravity, but a great amount of them suspend in air and diffuse along the airflow (Fig. 2B4; Supplementary Movie 2). Similarly, the fluid within the mouth can be slapped frequently as tongue swiping across the hard palate and other oral surfaces (e.g. teeth) in our eating or drinking, and result in a large amount of aerosol. During swallowing, the bolus flow is a turbulent flow (Chen 2009) with a velocity reaching as high as 0.5 m/s and an estimated Reynolds number as high as 10,000 (Hasegawa et al. 2005). In this case, fluid splashing to form aerosol particles is a high possibility. However, turbulence and its resulting aerosol particles were not in our simulation.

In the shear-induced mechanism of bio-aerosol generation, the passage of high-velocity air stream over the mucus surface will generate wave-like disturbances which lead to aerosol creation. The velocity of airflow during normal breath was reported to be insufficient to generate shear-induced aerosols (Morawska et al. 2009; Scheuch 2020; Xu et al. 2022), and hence the shear-induced mechanism may not be essential during the oral actions of food consumption (Ni et al. 2015). However, the expiratory airflow immediately after the completion of swallowing (Brodsky et al. 2010; Steele 2015; Hopkins-Rossabi et al. 2019) has the potential to generate shear-induced aerosols, due to its high velocity. A large number of fine aerosol particles were generated by the reversed-air flow after the squeezing of the dropper (Fig. 2C1–C4; Supplementary Movie 3), which was used to simulate the flow in the oropharyngeal region after bolus swallowing. It is not certain how big the contribution of exhaled airflow is to the generation of oral aerosol particles, due to the limited understanding of air velocity after the completion of swallowing. However, as a comparison, a study revealed that respiratory aerosols during intensive exercise were 10-fold greater than that during quiet breathing (Mutsch et al. 2022), due to significantly increased pace of breath and air velocity. This implies that shear-induced oral aerosol particles could also become dominant during intense eating and particularly during fast swallowing.

It is worth noting that the generation of aerosols is a combined result of the energy input and the properties of the fluid, especially its rheological and surface properties, the above evidence can only approve in principle that eating or drinking provides sufficient conditions for the generation of fine aerosol particles (as illustrated in Fig. 2E). Their full potentials as aerosol-generating procedures are yet to be investigated with varied food systems, human oral physiology, and eating behaviors. Indeed, aerosol particles ranging from 10 to 0.1 µm have also been identified in situ (Fig. 2D1 and D2; Supplementary Movie 4), which is also better regarded as qualitative evidence. Capturing such particles is largely by chance and the density could be less representative and varied greatly among different observations (Jermy et al. 2021; Li et al. 2021; Mueller et al. 2021; Varga et al. 2022).

#### The oral–nasal migration of aerosol particles during drinking

**Fig. 3.**
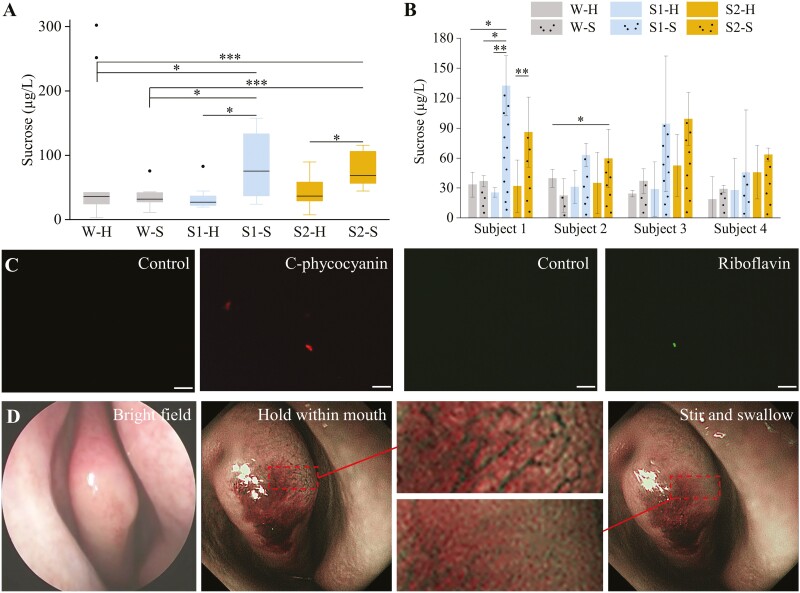
The oral–nasal migration of aerosol particles during drinking. A) Sucrose contents within the nostril-exhaled air compared across treatments. The nostril-exhaled air was collected with 3 samples (the pure water (W), 300 mM sucrose (S1), or 600 mM sucrose (S2)) under 2 oral behaviors (hold (H) or swirled and swallowed (S)), with 12 independently collected and analyzed samples in each condition. Box borders are 25th and 75th percentiles; bars are minimum and maximum value that calculated by 25th percentile − 1.5*interquartile or 75th percentiles + 1.5*interquartile; the value lower than the minimum value or higher than maximum value is determined as outlier (black dots); horizontal line is median. B) Sucrose contents within the nostril-exhaled air compared across subjects. C) Fluorescence images of the nostril-exhaled air deposited on the hydrophobic glass coverslips after the subjects consumed the pure water (control) or sample solution (800 mg/L c-phycocyanin or riboflavin aqueous solution). D) NBI of olfactory cleft when subject holding the c-phycocyanin solution within the mouth, and after swirling and swallowing the c-phycocyanin solution. Bright-field optical image of olfactory cleft was also obtained for comparison. All images were obtained from 1 subject during 1 trial. ***, **, and * represent the *P*-value of 0.001, 0.01, and 0.05.

The respiratory aerosol can be produced in large amounts, carrying both volatile and nonvolatile substances from the lungs and airways (Fennelly 2020) (e.g. virus (Ma et al. 2021), bacteria (Fennelly 2007), and volatile compounds (Cassagnes et al. 2021) to the outside of the human body, and cause airborne transmitted diseases like COVID-19 (Wang et al. 2021). Oral consumption of bottled pure water (W-H and W-S) gives similar sucrose contents (29.55 ± 13.41 µg/L with 2 outliers or 31.44 ± 9.21 µg/L with 1 outlier) in the collected samples; meanwhile, holding 300 (S1-H) or 600 mM (S2-H) sucrose solutions in mouth and spitting it out also gives close levels of sucrose content (28.04 ± 8.52 µg/L with 1 outliers or 41.21 ± 23.51 µg/L) to that of bottled pure water group (Fig. 3A). Although it is to our surprise that sucrose can be detected when the subjects consuming even bottled pure water, the close sucrose contents in W-H, W-S, S2-H, and S1-H assured us that this amount of sucrose is native in human’s nostril-exhaled air and simply swirling and swallowing the water did not increase it. Swirling and swallowing the 300 (S1-S) or 600 mM (S10-S) sucrose solution can give significantly higher sucrose contents (83.88 ± 48.13 and 77.09 ± 26.36 µg/L) (Fig. 3A), indicating the role of aerosol in the mass transfer of the sucrose from the oral cavity to the nasal cavity. Take the 300 mM sucrose as an example, the drinking actions can carry 2.4 × 10^−11^ mol sucrose within each nostril-exhaled air after the completion of swallowing, which equals 4.8 × 10^6^ aerosol particles with a particle size of 0.4 µm in diameter, an impressively high number of aerosol particles compared with the reported numbers for respiratory aerosols using particle counter (Edwards et al. 2021).

Surprisingly, the sucrose content in nostril-exhaled air of 600 mM sucrose solution (S-2S in Fig. 3A) was no higher than that of 300 mM sucrose solution (S-1S in Fig. 3A), indicating that further increased sucrose concentration did not favor the production of aerosol particles inside the oral cavity and/or their diffusion from the oral cavity to nostril. While the exact reason behind this observation is not clear, one may speculate that a higher viscosity and surface tension of the sucrose solution may have impeded the creation of oral aerosol particles. When these data were grouped by the subjects (Fig. 3B), similar trends were also observed that S1-S and S2-S were higher than other treatments which are close to each other, while their statistical differences were not obtained in certain cases probably due to the small sample sizes.

To further explore the potential of oral aerosol as carriers for substances with varied properties, fluorescence imaging was used to detect the appearance of riboflavin and c-phycocyanin in the nostril-exhaled air after the subjects consumed their solutions. Riboflavin and c-phycocyanin were selected because of their fluorescence and nonvolatilities. C-phycocyanin or riboflavin was detected in subjects’ nostril-exhaled air after oral consumption of c-phycocyanin or riboflavin solution; while no fluorescence was detected after the bottled pure water was consumed instead (Fig. 3C). It should be noted that c-phycocyanin or riboflavin can only emit moderate or low characteristic red or green fluorescence under green or blue light laser (Hou et al. 2017; Jermy et al. 2021) and aerosol particles could evaporate and merge rapidly once exposed to an external environment of low relative humidity (Mürbe et al. 2021), and hence only a few dots with sufficient size and concentration of c-phycocyanin or riboflavin can be observed here. The detection of c-phycocyanin, riboflavin, and sucrose in the nostril-exhaled air approved that aerosol particle has the capacity of migrating from the oral to nasal cavity, and this is true across various compounds.

To explore the possibility of aerosol deposition on olfactory cleft, NBI was applied based on the principle that the hemoglobin in the blood vessel can strongly absorb the narrow-band illumination (540 and 415 nm), making the blood vessel darker in NBI observation than the surrounding tissues and therefore a clear contrast can be observed (Valletti et al. 2019). Based on this principle, the deposition of fluorescent materials with similar absorbing characteristics (e.g. c-phycocyanin) on the olfactory cleft surface would also display a darker color and significantly weaken the contrast of blood vessels, and hence the surface deposition of such compounds can be identified. Subjects with a flexible endoscope positioned in their nostrils were asked to consume c-phycocyanin solution in their habitual manners. Results showed that simply holding the c-phycocyanin solution inside the oral cavity did not alter the fluorescence behavior of the olfactory cleft (Fig. 3D): the invisible blood vessel in the bright-field imaging was identifiable in the NBI. However, the blood vessels showed reduced contrast after the subject swirled and swallowed the c-phycocyanin solution (Fig. 3D), indicating the adsorption and/or deposition of a strong fluorescence material. It is most likely that the orally ingested c-phycocyanin is embedded in the oral aerosol created during drinking, and diffuses and deposit onto the nasal mucus around olfactory cleft, which makes the retro-nasal sensation of tastes factually possible.

Taking together, it is assured that aerosol can be produced, migrate and deposit onto the olfactory receptors during drinking, giving them the potential to be the alternative mechanism independently from the molecule.

## Experiment 2: the smelling of taste compounds

### Method

#### Study design

**Fig. 4.**
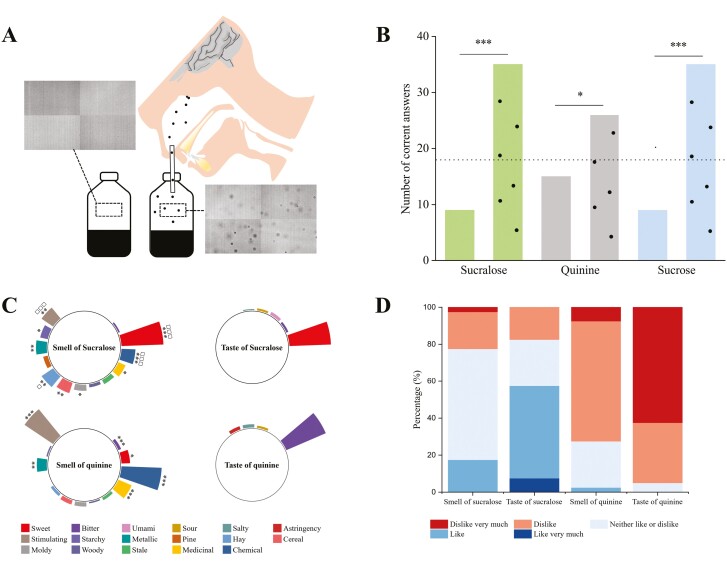
The ortho-nasal sensation of taste compounds within aerosol particles. A) Schematic illustration of the sensory tests. Headspace air of bottles 2 cm above their liquid surface after they stood still for more than 30 min or shaken upside down once and holds for 5 s were imaged. 100 mL tastant solution was in the 400 mL bottles. Scale bar = 100 µm. B) Number of correct answers out of 40 responses obtained from a test of 4AFC. The headspace air of the sucralose (20 mM), quinine (20 mM), or sucrose (300 mM) solution either stood still (bars) or shaken (bars with black dots) were sniffed via a straw and discriminated among the other 3 pure water by 40 subjects. A confidence level of 99.5% is met at 18 correct responses (dashed line). ***, **, and * represent the *P*-value of 0.001, 0.01, and 0.05. C) CATA results of the smell or taste of sucralose or quinine solution. ***, **, and * represent the *P*-value of 0.001, 0.01, and 0.05 between the smell and taste, and □□□ and □ represent the *P*-value of 0.001 and 0.05 between the smell of sucralose and quinine. D) The liking rate of the smell or taste of sucralose or quinine solution.

**Fig. 5.**
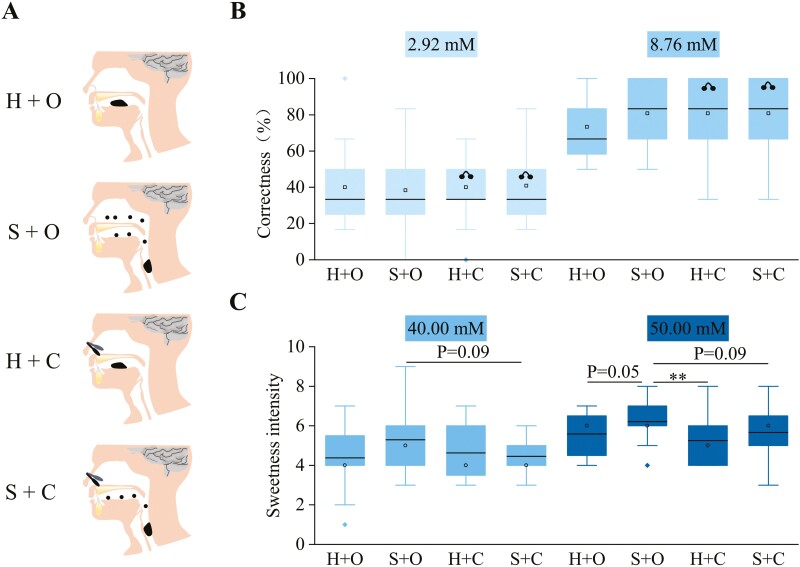
The retro-nasal sensation of taste compounds during drinking. A) Schematic illustration of 4 conditions used in the retro-nasal evaluation tests. H and S refer to 2 oral processing condition that were holding the solution for 5 s and spitting it out and swirling the solution for 5 s and swallowing it. O and C refer to 2 nasal conditions that were naturally open and clipped. Black dots represent the aerosol particles. B) The correctness of 20 subjects in a test of 3AFC under 4 different conditions (H + O, S + O, H + C, or S + C). 0.10% and 0.30% sucrose solution were tested. C) The intensity rating of sucrose solution obtained from 40 subjects. Significance was determined using ANNOVA Dunnett’s post hoc tests. Box borders are 25th and 75th percentiles; bars are minimum and maximum value that calculated by 25th percentile − 1.5*interquartile or 75th percentiles + 1.5*interquartile; the value lower than the minimum value or higher than maximum value is determined as outlier (black dots); horizontal line is median. ** represents the confidence levels (*P*) of 0.01.

The purpose of this study was to explore whether the nonvolatile taste compounds can be smelled in the form of aerosol during food consumption. In general, the ortho-nasal sensation of air with or without the in vitro prepared aerosol particles was determined to assure whether the taste compounds themselves can arouse olfaction (Fig. 4A); while, the retro-nasal sensation of taste compounds during drinking was determined with or without the nose-clip to explore the potential involvements of olfaction (Fig. 5A). The in vitro prepared aerosol particles were tested via the ortho-nasal route, instead of retro-nasal route, was to prevent the possible oral taste sensation.

#### The ortho-nasal sensation of taste compounds within aerosol particles

Untrained healthy subjects (20 males and 20 females, average ages of 23.6 ± 1.1, BMI of 20.5 ± 1.6) that were naive to the purposes of the study and with no self-reported anosmia were recruited for this experiment. Two different tasks were designed to approve the potential ortho-nasal sensation of taste compounds. In the first part, subjects were asked to indicate which of the four 400 mL bottles (3 with 100 mL bottled pure water and 1 with 100 mL taste solution) smelled differently from the other 3 after sniffing their headspace air via straws, which is a four-alternative forced choice (4AFC) paradigm. In each test, all 4 bottles either stands still for at least 10 min or were shaken up and down for once and stood still for 5 s before being sniffed. Sucrose, quinine hydrochloride (98%, Aladdin Inc., China), and sucralose (98%, Aladdin Inc., China) were selected as the representative nonvolatile taste compounds and were dissolved in bottled pure water at the concentrations of 200, 20, and 20 mM, respectively. In the second part, subjects were asked to pick all the attributes they felt after orally taste the sucralose (0.2 mM), quinine solution (0.1 mM), or ortho-nasally “smell” their aerosol particles, which is a check-all-that-apply (CATA) test. The listed attributes were sweet, bitter, umami, sour, salty, astringency, stimulating, starchy, metallic, pine, hay, cereal, moldy, woody, stale, medicinal, and chemical, which were generated by 4 trained panelists. Liking was also rated by the subjects. The aerosol particles created by the shaking of bottles were visually approved via the abovementioned high-speed camera through the holes that are 1 cm above the liquid surface.

#### The retro-nasal sensation of taste compounds

Two nasal and oral processing conditions were designed (Fig. 5A): the 2 nasal conditions were the nasal cavity fully open which allows aerosol particles to reach the olfactory epithelium through the oro-nasal pharynx during exhalation (O), and nasal cavity fully blocked by using nose-clipped (C), which prevented aerosol particles from reaching the olfactory epithelium; the 2 oral processing conditions were holding the sucrose solution for 5 s and spitting it out (H) (no-sucrose aerosol generation), and swirling the sucrose solution for 5 s and swallowing it (S) (sucrose aerosol generation). 20 healthy subjects (10 males and 10 females, with average ages of 24.8 ± 0.77 and BMI of 20.3 ± 3.5) were asked to taste 3 cups (2 were 5 mL bottled pure water and 1 was 5 mL, 2.92- or 8.76-mM sucrose solution) in the required nasal and oral processing condition, and select the odd one. This procedure was carried out 6 times and the correctness were calculated and compared. The taste intensity of sucrose solution with moderate sweetness (40.00 or 50.00 mM) was also rated by 25 healthy subjects (14 males and 14 females, average ages of 24.8 ± 0.77, BMI 20.3 ± 3.5) in the required nasal and oral condition. All subjects were untrained and naive to the purposes of this experiment and have no self-reported anosmia.

#### Statistics and significance

The possibility of the taste compounds being detected in the task of 4AFC in Study design was determined using the binomial distribution: A confidence level of 99.5% is met at 18 correct responses. The differences between stood or shaken bottles in the 4AFC and attributes in CATA results in Study design were analyzed using chi-square test. The differences between the treatments (H + O, S + O, H + C, and S + C) in the 3AFC and taste intensity results of The retro-nasal sensation of taste compounds were analyzed using ANNOVA Dunnett’s post hoc tests.

### Results

The smelling of taste compounds is still controversial. Although healthy subjects were reported to distinguish the pure taste solution from water by simply sniffing the air above them, and perceived a significantly reduced intensity of taste solution when their noses were clipped (Mojet et al. 2005), it is still debated whether these were caused by the trace amount of volatile contaminants (Stevenson and Attuquayefio 2013) or taste compounds themselves (Mojet et al. 2005). While the trace amount of volatile contaminants is unlikely to impose substantial retro-nasal sensation, it is also conflicted with the current belief that only volatile compounds can be accessed and sensed by the olfactory receptors during drinking or eating. In our observations, subjects cannot distinguish the taste solution from bottled pure water, by sniffing the headspace of bottles that stand still (Fig. 4A and B). Therefore, the volatile compound did not exist or at least below the threshold of smell detection in the taste solutions we measured. After being shaken, aerosol particles with varying sizes were observed (Fig. 4A) in the headspace of these bottles, and the taste solutions can all be distinguished from bottled pure water (*P* < 0.01) (Fig. 4B). The chances of the differences between standing and shaking were too small to be considered as mere coincidences (*P* < 0.05 for quinine, and *P* < 0.001 for sucrose and sucralose). The smell of aerosol particles (sucralose or quinine solution) is quite different from their oral taste (Fig. 4C), indicating the smell is not solely obtained from the back of oral cavity and throat after their deposition; while the smell of sucralose is also quite different from quinine (Fig. 4C), indicating the smell is not solely a nasal physical stimulation. The overall liking is also quite different among each other (Fig. 4D).

Since shaking the bottles can create smellable aerosol particles, it is possible that drinking actions can also create retro-nasally smellable aerosol particles. Nose-clip did not significantly reduce the detection of sucrose at the concentration around threshold (Fig. 5B). The concentration of sucrose might be too dilute to produce significant olfaction. At higher concentration, the nose-clip have a tendency to reduce the perceived sweetness intensity (*P* = 0.09) in the condition of swirling and swallowing (S + O), while no effects were observed in the condition of holding and spitting out (H + O). It is likely that drinking actions (swirling and swallowing) can create aerosol particles just like shaking the bottle, and these aerosol particles can migrate into the olfactory epithelium and arouse olfaction. However, the actual sensory contribution of the retro-nasal sensation might not be evaluated appropriately by the sweetness intensity, as many subjects in our study reported a loss of sensory experiences during the swallowing after their nose being clipped, while not sure whether it can be interpreted as reduced sweetness intensity. This is consistent with the reports (Mozell et al. 1969; Masaoka et al. 2010) that blocking retro-nasal sensation can significantly reduce the identification of sucrose solution. More sensory evidence will be collected in our following studies.

## Experiment 3: the relative efficiency of aerosol mechanism

### Method

#### Study design

**Fig. 6.**
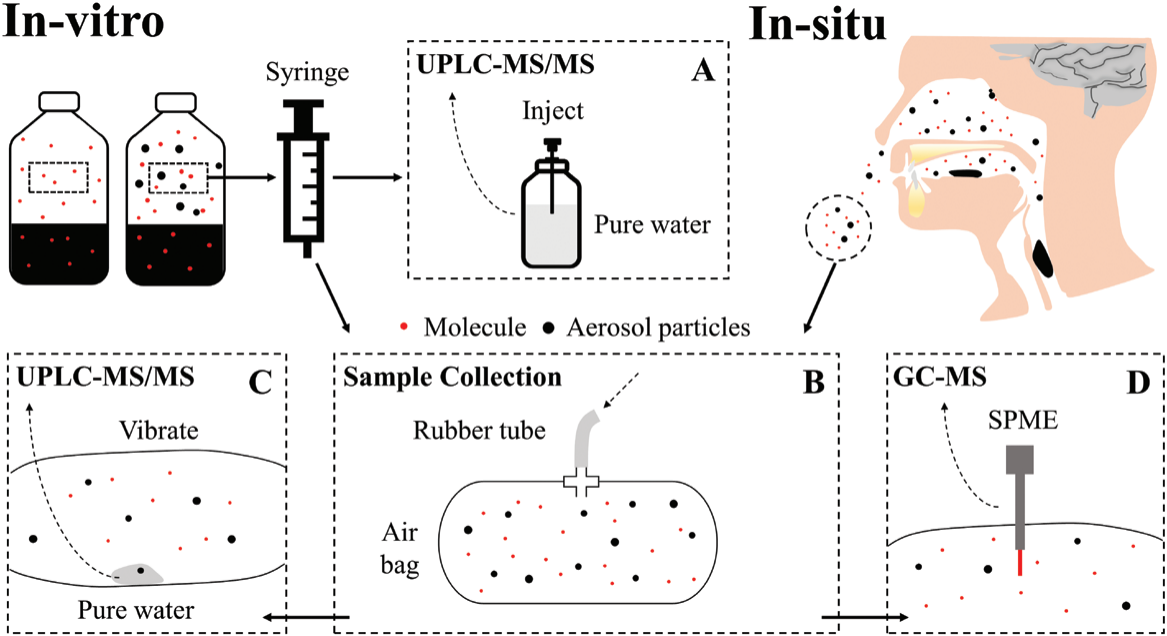
Schematic illustration of experiment 3. In vitro and in situ collected samples were illustrated, when volatile and nonvolatile coexisted in bottles or oral cavities. A) UPLC–MS/MS analysis of the headspace air. B) Sample collection for nostril-exhaled air and headspace air of bottles. C) UPLC–MS/MS analysis of the nostril-exhaled air. D) GC–MS analysis of nostril-exhaled air and headspace air of bottles.

The purpose of this study was to measure the relative efficiency of aerosol as an alternative mechanism of oral–nasal mass transfer, which is also of critical importance. A mixed aqueous solution of 300 mM sucrose, 4.5 mM vanillin (99%, w/w, Aladdin, China), and 8 mM ethyl acetate (99.5%, w/w, Sigma-Aldrich, USA) were tested under the in vitro or in situ conditions, and their contents within the headspace air or nostril-exhaled were quantified and compared (Fig. 6).

#### UPLC–MS/MS of nostril-exhaled air and bottle headspace

**Fig. 7.**
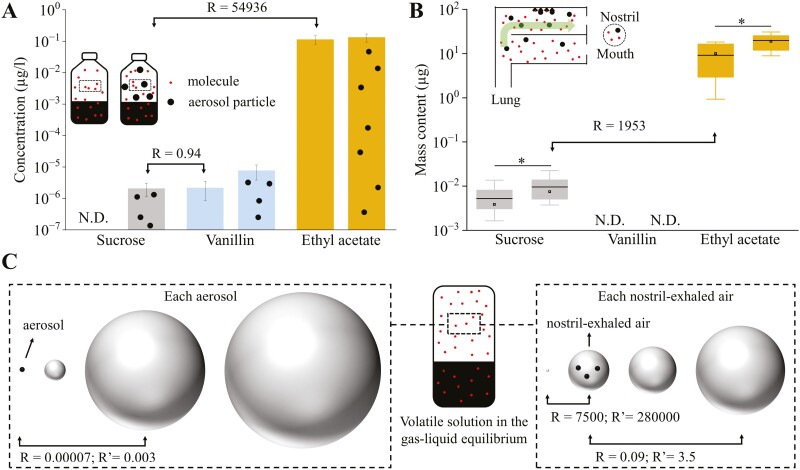
The efficiency of aerosol mechanism. A) Sucrose, vanillin, and ethyl acetate contents within the headspace of bottles stood still (bars) or shaken and hold for 5 s (bars with black dots) were quantified using UPLC–MS/MS and GC–MS. Three replicates were conducted for each condition, with a mixture solution of 300 mM sucrose, 8.1 mM ethyl acetate, and 4.5 mM vanillin. See “Method section” for the detailed collecting procedures. *P*-value = 0.06, 0.07, and 0.6 (from left to right). *R* = concentration ratios of vanillin or ethyl acetate to sucrose. B) Sucrose, vanillin, and ethyl acetate contents within the nostril-exhaled air when the subjects hold and spat out (left bars) or swirled and swallowed (right bars) the samples were quantified using UPLC–MS/MS and GC–MS. Twelve samples were collected and analyzed independently from 12 subjects in each condition, with a mixture solution of 300 mM sucrose, 8.1 mM ethyl acetate, and 0.4 mM vanillin. See “Method section” for the detailed collecting procedures. Box borders are 25th and 75th percentiles; bars are minimum and maximum value that calculated by 25th percentile − 1.5*interquartile or 75th percentiles + 1.5*interquartile; the value lower than the minimum value or higher than maximum value is determined as outlier (black dots); horizontal line is median. * represents the *P*-value of 0.05. *R* = concentration ratios of vanillin or ethyl acetate to sucrose. C) The volume of headspace air that was in their states of air–liquid equilibrium that needed to obtain the same molecule content of sucrose in each aerosol or each exhaled air. The calculation process and more comparisons are listed in Table 2. *R* = concentration ratios of vanillin or ethyl acetate (with liquid concentration of 4 mM) to sucrose. *R*ʹ = concentration ratios of vanillin or ethyl acetate (with liquid concentration of 300 mM) to sucrose.

In vitro *conditions*: Plastic bottles (400 mL maximum capacity) with 100 mL mixed aqueous solution were prepared the day before the test, sealed, and stored at 4 °C overnight. Return to room temperature (25 °C) before the test. 60-mL syringe was used to suction the headspace air of bottles either stands still for at least 10 min or were shaken up and down for once and stood still for 5 s, and inject the air into the bottles for the UPLC–MS/MS. Three replicates were conducted. The experimental procedure is illustrated in Fig. 6A and the results are listed as sucrose in Fig. 7A.

**Table 2.** Efficiency comparison between molecule and aerosol mechanism.

**Set 1. Constants**			**Set 2. Estimated values**		
	*M*	$K_{w}^{a}$	*V*_1_	*V*_2_	*C*_1_
Sucrose	3.4 × 10^2^	/	1.0 × 10^−1^	3.4 × 10^−17^	4.0 × 10^−3^
α-Terpinene	1.4 × 10^2^	1.4 × 10^0^	*C*_2_	*V*_*m*_	
Ethyl acetate	1.2 × 10^2^	6.8 × 10^−3^	1.5 × 10^−1^	2.2 × 10^1^	
Acetaldehyde	4.4 × 10^1^	3.1 × 10^−3^			
Methyl jasmonate	2.2 × 10^2^	5.8 × 10^−7^	**Set 3. Measured values**		
Vanillin	1.5 × 10^2^	8.6 × 10^−8^	*m*_1_	*m*_2_	
2-Ethylbutanoic acid	1.2 × 10^2^	4.1 × 10^−10^	5.1 × 10^−3^	1.0 × 10^−2^	
**Set 4. Calculated values**					
	*Y*_2_	${Y^{\prime }}_{2}$	*C*_3_	${C^{\prime }}_{3}$	*Y*_1_
α-Terpinene	1.0 × 10^−4^	3.8 × 10^−3^	2.3 × 10^−3^	8.4 × 10^−2^	7.3 × 10^−5^
Ethyl acetate	5.0 × 10^−7^	1.8 × 10^−5^	1.1 × 10^−5^	4.1 × 10^−4^	
Acetaldehyde	2.3 × 10^−7^	8.4 × 10^−6^	5.1 × 10^−6^	1.9 × 10^−4^	
Methyl jasmonate	4.2 × 10^−11^	1.6 × 10^−9^	9.4 × 10^−10^	3.5 × 10^−8^	${Y^{\prime }}_{1}$
Vanillin	6.3 × 10^−12^	2.3 × 10^−10^	1.4 × 10^−10^	5.2 × 10^−9^	2.7 × 10^−3^
2-Ethylbutanoic acid	3.0 × 10^−14^	1.1 × 10^−12^	6.7 × 10^−13^	2.5 × 10^−11^	
	*n*_1_	*n*_2_	*C*_4_	*N*	
	1.5 × 10^−10^	5.0 × 10^−18^	1.5 × 10^−9^	3.0 × 10^7^	
**Set 5. Efficiency comparison**					
	*R*_1_	${R^{\prime }}_{1}$	*R*_2_	${R^{\prime }}_{2}$	
α-Terpinene	1.5 × 10^−2^	5.6 × 10^−1^	1.5 × 10^6^	5.7 × 10^7^	
Ethyl acetate	7.4 × 10^−5^	2.8 × 10^−3^	7.5 × 10^3^	2.8 × 10^5^	
Acetaldehyde	3.4 × 10^−5^	1.3 × 10^−3^	3.4 × 10^3^	1.3 × 10^5^	
Methyl jasmonate	6.3 × 10^−9^	2.3 × 10^−7^	6.3 × 10^−1^	2.3 × 10^1^	
Vanillin	9.3 × 10^−10^	3.5 × 10^−8^	9.4 × 10^−2^	3.5 × 10^0^	
2-Ethylbutanoic acid	4.4 × 10^−12^	1.6 × 10^−10^	4.5 × 10^−4^	1.7 × 10^−2^	

*M* = molecular mass (g/mol); $K_{w}^{a}$ = water–air phase equilibrium constant; *V*_*m*_ = molar volume of air (L/mol); *N* = number of aerosol in each nostril-exhaled air; *V*_1_ = volume of one exhaled air after swallowing or spitting out of solution (L); *V*_2_ = volume of one aerosol with a radius of 0.2 µm (L); *C*_1_ = mass concentration of volatile compounds in volatile compounds–saliva mixture (mol/L); *C*_2_ = mole concentration of sucrose in sucrose–saliva mixture (mol/L); *C*_3_ or ${C^{\prime }}_{3}$ = calculated mole concentration of volatile compounds in headspace air (mol/L); *C*_4_ = mole concentration of sucrose in nostril-exhaled air (mol/L); *m*_1_ or *m*_2_ = detected sucrose contents in the nostril-exhaled air that following holding and spitting or swirling and swallowing sucrose solution (µg); *n*_1_ or *n*_2_ = mole content of sucrose within each nostril-exhaled air or aerosol (mol); *Y*_1_ or ${Y^{\prime }}_{1}$ = mole fraction of volatiles in liquid at a concentration of *C*_1_ or *C*_2_; *Y*_2_ or ${Y^{\prime }}_{2}$ = mole fraction of volatiles in headspace air at a concentration of *C*_1_ or *C*_2_; *R*_1_ or ${R^{\prime }}_{1}$ = ratios of volatile compounds to sucrose per aerosol at concentration of *C*_1_ or *C*_2_; *R*_2_ or ${R^{\prime }}_{2}$ = ratios of volatile compounds to sucrose per breath at a concentration of *C*_1_ or *C*_2_.

In situ *conditions*: The sucrose contents within the nostril-exhaled air of 12 healthy subjects (6 males and 6 females, average ages of 23.7 ± 0.5, BMI of 20.6 ± 1.7) were collected and analyzed in the same way as mentioned in UPLC–MS/MS of nostril-exhaled air. The experimental procedure is illustrated in Fig. 6B and C, and the results are listed as sucrose in Fig. 7B.

#### Gas chromatography–mass spectrometry of nostril-exhaled air and bottle headspace

In vitro *conditions*: The headspace air of bottles was collected the same way in the UPLC–MS/MS, and inject the air into the airbags. The vanillin and ethyl acetate within the airbags were extracted with solid-phase microextraction fiber (SPME), and then quantified with gas chromatography–mass spectrometry (GC–MS; see Supplementary Information for the details). Three replicates were conducted. The experimental procedure is illustrated in Fig. 6B and 6D and the results are listed as vanillin and ethyl acetate in Fig. 7A.

In situ conditions: The vanillin and ethyl acetate within the nostril-exhaled air of 12 healthy subjects (6 males and 6 females, average ages of 23.7 ± 0.5, BMI of 20.6 ± 1.7) were collected in airbags in the same way as mentioned in UPLC–MS/MS of nostril-exhaled air, and were extracted with SPME, and then quantified with GC–MS (see Supplementary Information for the details). The experimental procedure is illustrated in Fig. 6B and D and the results are listed as vanillin and ethyl acetate in Fig. 7B.

#### Efficiency comparison between aerosol and molecule mechanisms

The sucrose concentration (*C*_2_) in each nostril-exhaled air following the swallowing of 5 mL 10% sucrose solution can be obtained as


$$\begin{array}{l} C_{2}=\frac{m_{1}-m_{2}}{V_{1}*M_{Sucrose}} \end{array}$$ (1)

where *m*_1_ or *m*_2_ are the detected sucrose contents in the nostril-exhaled air following the holding and spitting or swirling and swallowing sucrose solution (data plotted in Fig. 5B were used here). *M*_sucrose_ is the mole mass of sucrose. The sucrose concentration within one aerosol (*C*_1_) equals the concentration of sucrose in saliva-sucrose mixtures. The efficiency comparison between aerosol and molecule mechanisms per aerosol (*R*_1_) or per nostril-exhaled air (*R*_2_) was calculated as


$$\begin{array}{l} R_{1}=\frac{C_{1}}{C_{3}},\;R_{2}=\frac{C_{2}}{C_{3}} \end{array}$$ (2)

where *C*_3_ is the concentration of volatile compounds in the headspace air with air–aqueous equilibrium. The liquid concentrations of volatile compounds were set to 500 mg/kg (the same as the concentration of ethyl acetate in saliva–mixed solution mixtures), and 0.15 mol (the same as the concentration of sucrose in saliva–sucrose mixtures), for comparison. *C*_3_ can be calculated from Henry’s law,


$$\begin{array}{l} C_{3}=\frac{Y_{2}}{V_{m}},\;\;Y_{2}=K_{w}^{a}*Y_{1} \end{array}$$ (3)

where *Y*_2_ is the mole fraction of volatiles in the air phase, *V*_*m*_ is the molar volume of air, $K_{w}^{a}$ is the water–air phase equilibrium constant, and *Y*_1_ is the mole fraction of volatiles in the water phase. The constants, measured, estimated, and calculated values used or produced in the calculation are listed in Table 2.

#### Statistics and significance

The differences between stood or shaken bottles (Fig. 7A) or treatments (Fig. 7B) within each tested compound were analyzed using 2-tailed paired-samples *T* test.

### Result

The relative efficiency of the aerosol mechanism is calculated in 3 different methods: the sucrose contents that were experimentally detected in the headspace of bottles or nostril-exhaled air, were compared with the volatile compounds that were also experimentally detected in the headspace of bottles (Fig. 7A) or nostril-exhaled air (Fig. 7B), or theoretically calculated (Fig. 7C).

Only the volatile compounds (vanillin and ethyl acetate) can be detected when bottles stand still (bars in Fig. 7A), while the sucrose was also detected when the bottles were shaken (bars with black dots in Fig. 7A). This was consistent with our findings in Fig. 4B that nonvolatile taste compounds can be smelled after their solutions being shaken. The sucrose concentration in the headspace air of shaken bottles was close to that of vanillin in bottles that stood still (the vanillin/sucrose ratio, *R*, is 0.94), indicating the relative efficiency of aerosol particle could be 1.6% of the released molecules with a volatility of vanillin. The vanillin concentration tripled after the bottles were shaken, the other hand, indicating the relative efficiency of aerosol particles could even outdo the released molecules. The aerosol particles generated by shaking make no difference in the ethyl acetate concentration in the headspace air, and their detected content is 54,936 times higher than the detected sucrose content. It is worth noting that the syringe we used to collect aerosol particles or molecules suspended within the air, and the collecting procedures might be more favorable toward the molecules and volatile compounds, hence the actual efficiency of aerosol particles should be higher than we calculated here.

Indeed, the sucrose and ethyl acetate contents within the nostril-exhaled air were closer than they in the headspace of shaken bottles (the ethyl acetate/sucrose ratio is 1,953 in Fig. 7B and 54,936 in Fig. 7A). Consistent with the results in Fig. 3, swirling and swallowing the solution (right gray bar in Fig. 7B) gave a significantly higher amount of sucrose than that in the group of holding and spitting out (left gray bar in Fig. 7B). It is interesting that action of swirling and swallowing also gave a 2.4 times ethyl acetate contents in nostril-exhaled air of that in the action of holding and spitting out (Fig. 7B). Currently, it is difficult to reason about the contribution of aerosol mechanism in ethyl acetate’s higher contents, since higher oral and pharynx exposure time during swirling and swallowing might also contribute.

Taking α-terpinene, ethyl acetate, acetaldehyde, methyl jasmonate, vanillin, and 2-ethylbutanoic acid as the representatives of high- or low-volatile aroma compounds, Fig. 7C and Table 2 gave us the idea of the relative concentration of sucrose in the form of aerosol. All the volatile compounds were calculated at the same concentration of sucrose (150 mM) or ethyl acetate (4 mM) in the saliva–solution mixtures, with the assumption that saliva incorporation is 1:1 with solution, and gas–liquid equilibrium is achieved. Since aerosol particle is directly the fractured part of saliva–sucrose mixture, its sucrose content equals that of the mixture, and hence is much denser than the aroma compounds released in the headspace air, even with high volatility as α-terpinene and ethyl acetate (*R* or *R*ʹ = 0.00007 or 0.03) (Fig. 7C). The high dense of aerosol might create a unique and strong retro-nasal sensation. Within each nostril-exhaled air, the concentration of ethyl acetate is 7,500 times of sucrose (Fig. 7C), which is between in vitro (Fig. 7A, *R* = 54,936) and in situ (Fig. 7B, *R* = 1,593) measured results. The theoretically calculated concentration ratio between vanillin and sucrose aerosol is much higher than the in vitro detected one (0.09 versus 0.94).

Taken together, the overall efficiency of the aerosol mechanism is within a reasonable range that potentially carries a substantial amount of olfactory substances from the oral to nasal cavity (1,000 to 1.6% of the molecule mechanism of vanillin, and 0.0000002%–0.001% of the molecule mechanism of vanillin), and its unique high regional dense might arouse unique retro-nasal sensation. However, the oral–nasal cavity remains largely a black box to us, and the trajectory of aerosol particles within it is also quite unknown, it is therefore hard to accurately estimate the relative efficiency of aerosol mechanism based on the nostril-exhaled air. Besides, the actual number of olfactory substances deposited onto the olfactory receptors should have a more direct relationship with their retro-nasal sensation and are co-influenced by the surface and fluid properties of both food and saliva, the migration and deposition behaviors of oral aerosol, the sensitivity of olfactory receptors and olfactory substances, etc., which are beyond the scope of this work and yet to be studied.

## Discussion

Aiming to explain a long-existing observation (Mozell et al. 1969; Mojet et al. 2005; Masaoka et al. 2010; Tsuji et al. 2018) that retro-nasal sensation seems to be involved in the oral consumption of nonvolatile taste compounds, we proposed aerosol as the alternative oral–nasal mass transfer and sensation mechanism operating independently from and also in parallel with the molecule mechanism during drinking and eating. Three immediate and critical concerns are tested here regarding the plausibility of this mechanism: (i) can aerosol particles be generated and migrate within the oral–nasal tract during drinking or eating; (ii) can taste compounds within aerosol particles be smelled; (iii) can aerosol particles be substantial carriers of olfactory substances?

The in situ evidence (Figs. 3 and 7) collected from different perspectives can approve in principle that the aerosol particle can be generated and migrate during drinking. However, all the results were based on the aqueous solution with relatively small sample sizes, hindering us from making a generalized conclusion across the varied food systems, human oral physiologies, and eating behaviors. The in vitro simulation (Fig. 2) can only approve in principle that certain oral and pharyngeal actions during food consumption have the potential to generate aerosol particles, the visualization of in situ aerosol generation and migration is still needed to understand what really happen within the oral–nasal cavity. Currently, it is still a huge challenge to image the small optical aqueous particles (<1 µm) moving at relative high speed within the oral–nasal cavity.

The proposed aerosol mechanism might also blur the believed strict boundary between smell and taste of human-beings that the nonvolatile taste compounds can also travel within the air when they are embedded in the aerosol particles, and therefore support the concept of unichemosensation proposed recently by Mollo et al. (2022). Many un- or not well-explained experimental observations could therefore be reasonably explained by the retro-nasal sensation of aerosol. For example, the perceived taste intensity was significantly reduced after the subjects’ noses were clipped as observed in our study (Fig. 5) and others (Mojet et al. 2005), and the recognition and detection thresholds of taste compounds were also significantly increased after the subjects’ noses were clipped (Tsuji et al. 2018). However, we acknowledged that eating or drinking perception is a very composite result of gustation, olfaction, hearing, touch, sight, personal psychology, and physiology, the untrained or even trained panelists cannot aware of the initiation of retro-nasal sensation and distinguish it from the gustation during drinking and eating, and hence their actual contribution is not identified. For example, blocking the retro-nasal sensation can significantly reduced the subjects’ identification of sucrose solution (Mozell et al. 1969; Masaoka et al. 2010), while the missing information is not revealed.

In our calculation, the aerosol mechanism can even be more efficient than the molecule mechanism for the low-volatile compounds (Fig. 7 and Table 2). However, this calculation might over- or under-rate the olfactory contribution of aerosol particles considering the limited understanding of the trajectory of aerosol particles and molecules. Besides, this calculation is based on the aqueous solution of sucrose and certain volatile compounds, the actual contribution of aerosol mechanism among various food or beverage systems is yet to be investigated. As a comparison, the generation of respiratory aerosols was found to be sensitive toward the fluid properties of surface mucous: a high surface viscoelasticity resists stretching of the mucus surface and therefore reduces the possibility of aerosol formation; while a low surface tension lowers the energy needed for small droplet creation (Watanabe et al. 2007; Hamed and Fiegel 2014; Hamed et al. 2020). Therefore, it can be predicted that the changing composition and microstructure of the mucus layer at oropharyngeal surfaces during food consumption can significantly alter the generation of aerosol particles and its resulting olfactory perception.

The high concentration of olfactory stimuli within each aerosol is 1 unique characteristic of the aerosol mechanism. Each aerosol with a diameter of 0.4 mm and 150 mM sucrose can have around 3 × 10^6^ molecules, which is much denser than volatile compounds released in the air (Fig. 7C). The other unique characteristic is that each aerosol particle can give an instant release of all olfactory stimuli once it is adsorbed at olfactory surface, again much more efficient than molecule mechanism which requires re-equilibrium between the air phase and the mucus fluid. Therefore, an immediate high local concentration of olfactory stimuli can occur and hence lead to a sudden increase of sensory stimulation once aerosol particles deposit. As being well approved that the pulsed inputs of sensation with strong peaks can cause higher overall intensity perception no matter in gustation (Mosca et al. 2013; Hutchings et al. 2015; Kistler et al. 2021) or olfaction (Nakao et al. 2013a, 2013b), the aerosol is therefore promising in reducing the overall usage of food flavor or create a unique sensory perception with its instant deliver of a high amount of olfactory substances. Again, more evidence is needed to fill the knowledge gap in this application.

In summary, this study approved the generation, migration, deposition of aerosol particles, and their possible retro-nasal sensation, providing a plausible explanation toward the involvement of retro-nasal sensation during the drinking of nonvolatile taste compounds. In order to gain a more comprehensive understanding of aerosol mechanism as an alternative oral–nasal mass transfer and resource of retro-nasal sensation, the following information is necessary: (i) the visualization and quantification of the in situ and in vitro aerosol generation and migration process with varied food systems, oral physiologies, and eating behaviors; (ii) the quantification of the nonvolatile compounds deposited on olfactory cleft; (iii) the sensory contribution of the retro-nasal sensation of aerosol during drinking or eating; (iv) the more direct efficiency comparisons between the molecule and aerosol mass transfer.

## Supplementary Material

bjad028_suppl_Supplementary_Material

## Data Availability

Data will be made available on request.
