# Supplementary material for: Can nonvolatile tastants be smelled during food oral processing?
Source: Chem Senses. 2023 Aug 17;48:bjad028. doi: 10.1093/chemse/bjad028 (PMC10516591; doi:10.1093/chemse/bjad028)
Supplement: bjad028_suppl_Supplementary_Material [file bjad028_suppl_supplementary_material.zip › Supplementary information.docx]

1. **Experiential settings and results of UPLC-MS/MS**

An ultra-high performance liquid chromatography (Acquity UPLC H-Class, Waters inc., USA) coupled with tandem mass spectrometry (TQSmicro, Waters inc., USA) (UPLC-MS/MS) was used to quantify the sucrose content in the collected samples. The representative chromatography of sucrose solution, collected sample, the standard curve of sucrose and the specific setting of UPLC-MS/MS was displayed in supplementary fig. 1.


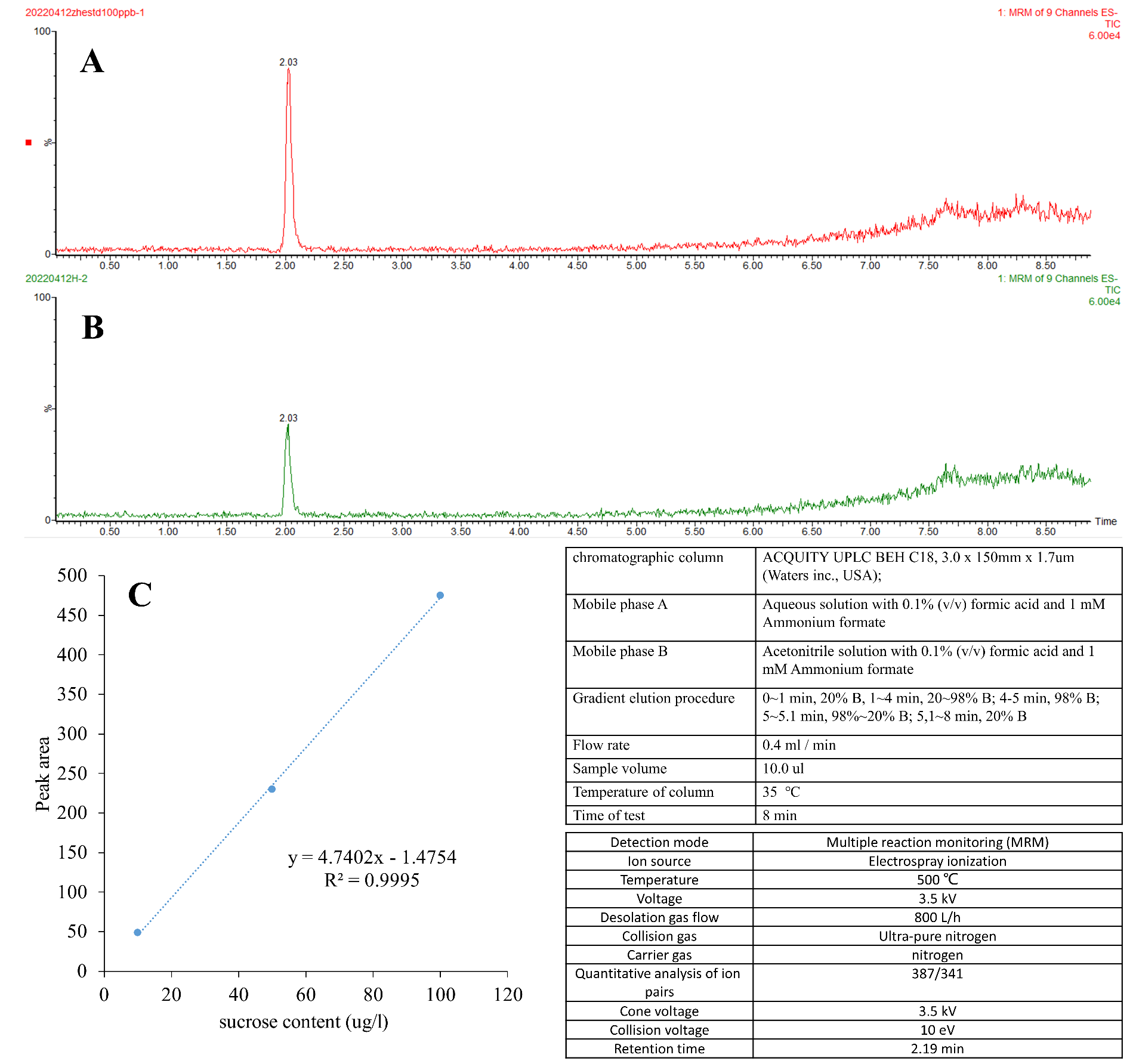


**Supplementary Fig. 1 Experimental setting and results of UPLC-MS/MS.** (A) chromatogram of standard sucrose solution (100 ppb), the characteristic peak at 2.03 min represents the sucrose. (B) chromatogram of one collected sample of nostril-exhaled air, the characteristic peak at 2.03 min represents the sucrose. (C) standard curve of sucrose solution. The tables were the parameters and settings of UPLC-MS/MS.

1. **Experiential settings and results of GC-MS**

A gas chromatography (Agilent 7890A, Agilent inc., USA) coupled with mass spectrometry (Agilent 5975C, Agilent inc., USA) (GC-MS) was used to quantify the ethyl acetate and vanillin content in the collected samples. The representative chromatography of vanillin, ethyl acetate, collected sample, the standard curve and the specific setting of GC-MSD was displayed in supplementary fig. 2.


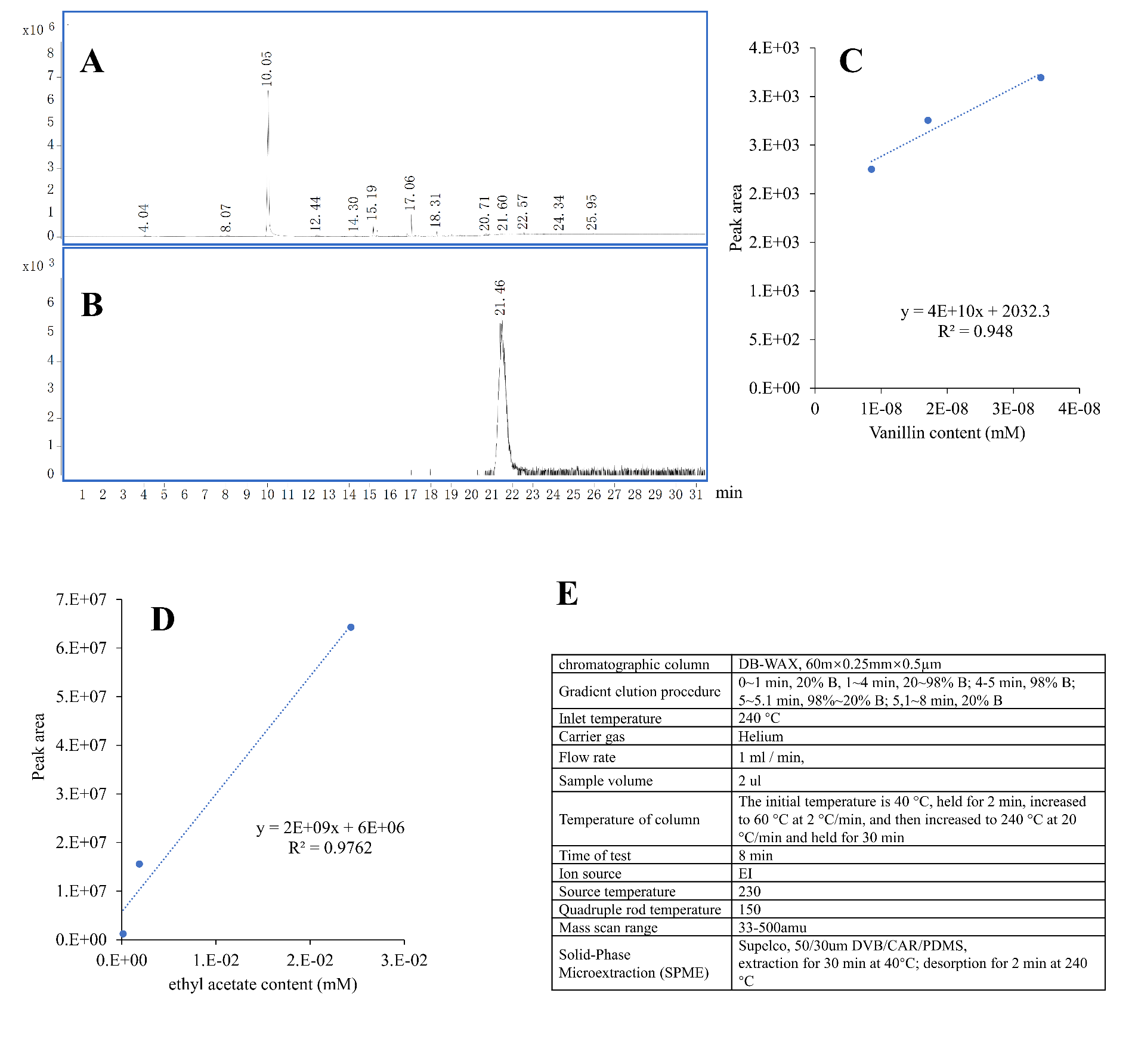
**Supplementary Fig. 2 Experimental setting and results of GC-MS.** (A-B) chromatogram of collected sample with characteristic peaks at 10.05 (A) and 21.46 (B) represent the ethyl acetate and vanillin. (C-D) The standard curve of vanillin (C) and ethyl acetate (D). (E) the parameters and setting of GC-MS.
