# Supplementary figures and images for: Can nonvolatile tastants be smelled during food oral processing?
Source: Chem Senses. 2023 Aug 17;48:bjad028. doi: 10.1093/chemse/bjad028 (PMC10516591; doi:10.1093/chemse/bjad028)

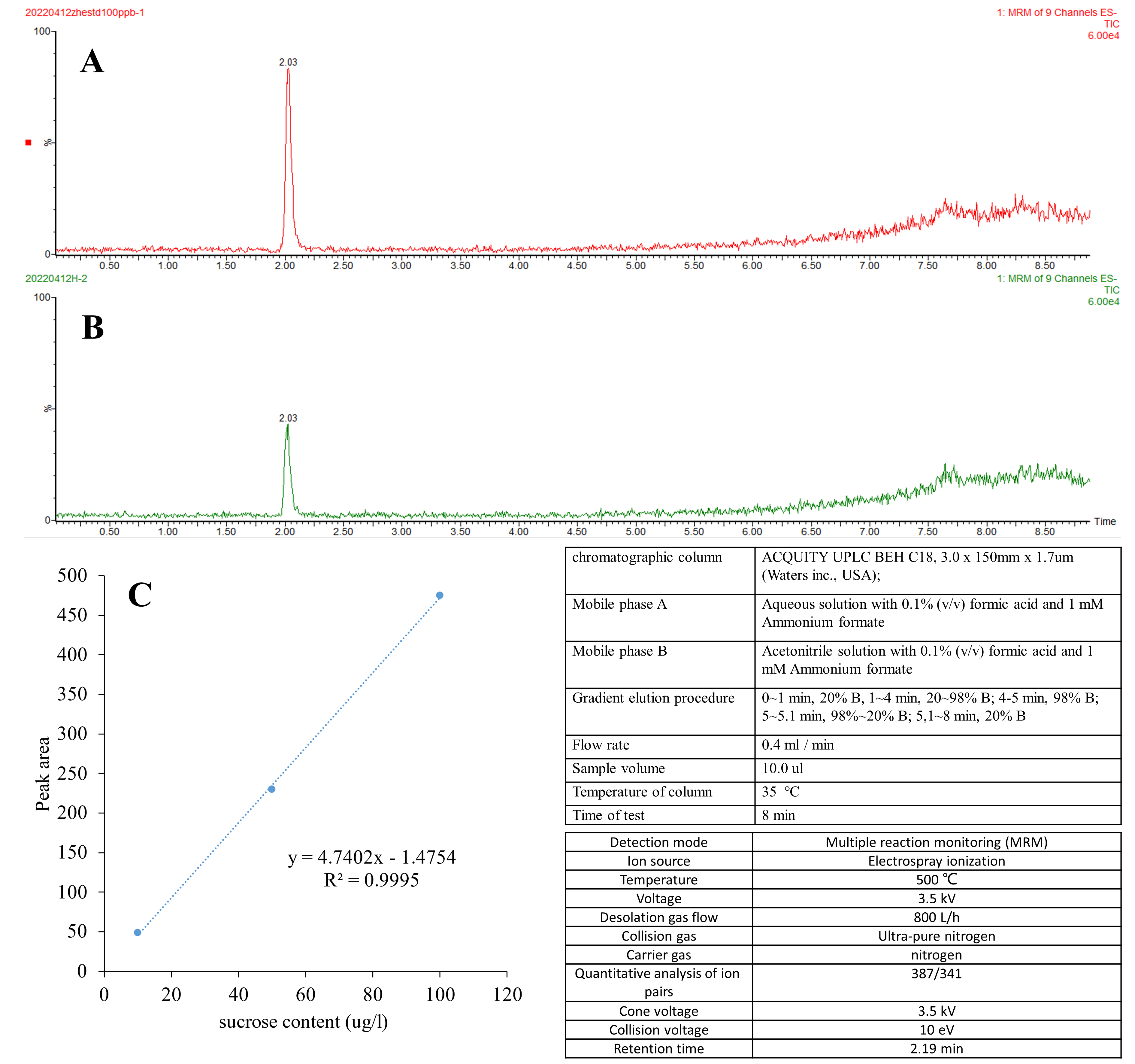

Supplement: bjad028_suppl_Supplementary_Material [file bjad028_suppl_supplementary_material.zip › S1.TIF]

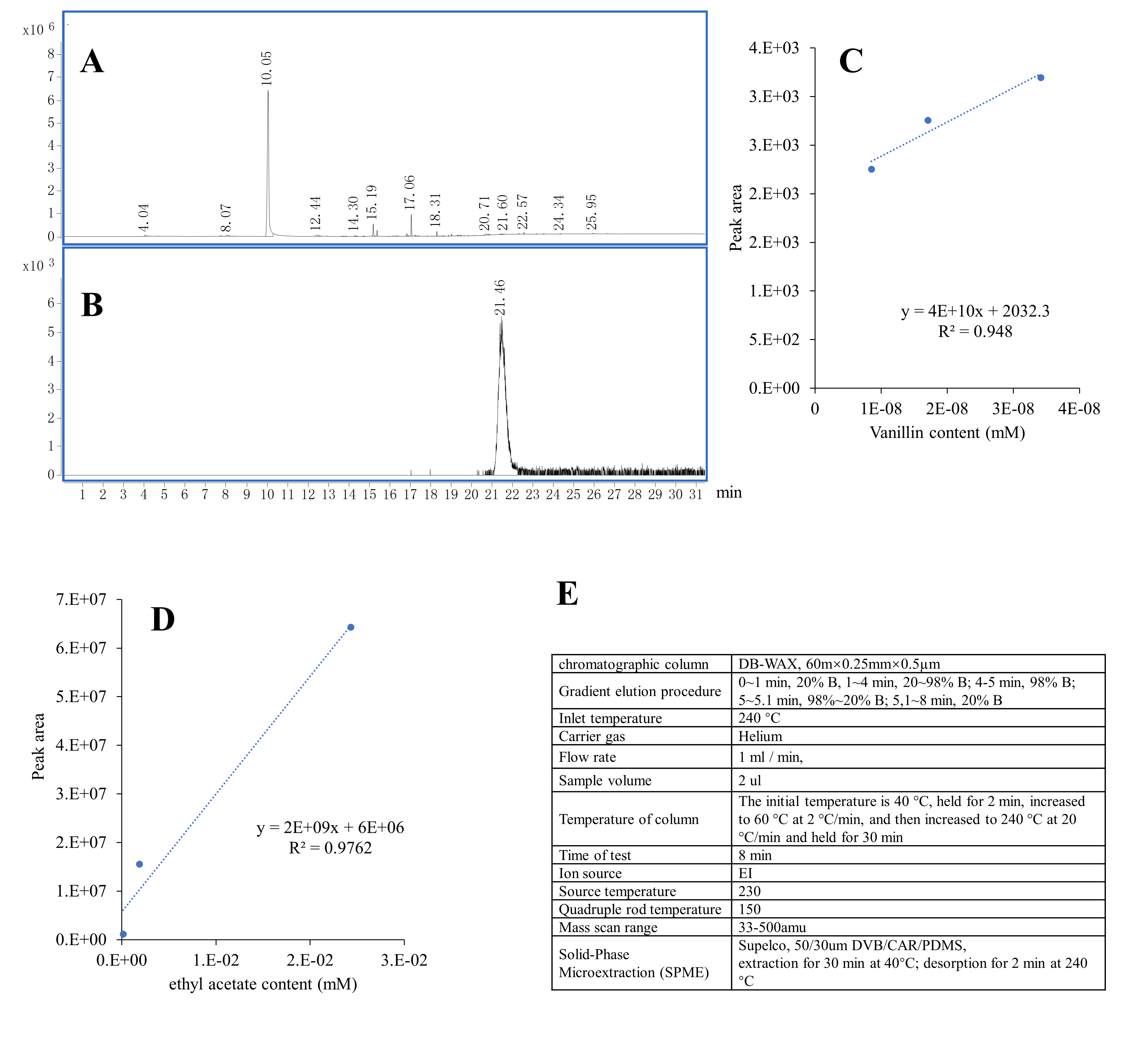

Supplement: bjad028_suppl_Supplementary_Material [file bjad028_suppl_supplementary_material.zip › S2.TIF]
